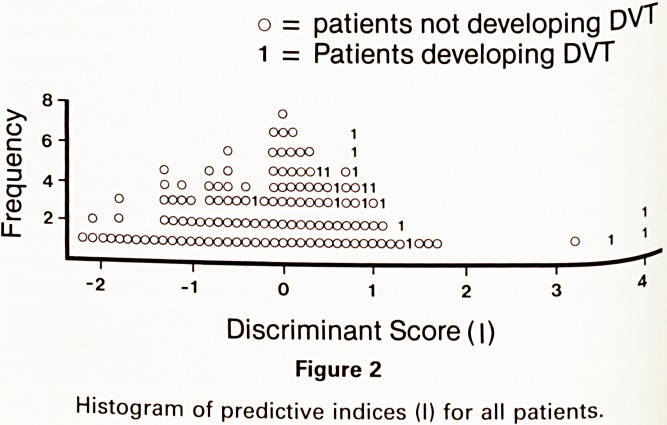# A Predictive Index for Postoperative Deep Vein Thrombosis in Thoracic Surgery Patients

**Published:** 1987-11

**Authors:** R B Richardson, C Dufresne, G E Staddon, F A Chudry, P Whitehead, K Jeyasingham

## Abstract

In a single-centre prospective trial 200 consecutive patients undergoing thoracic surgery were randomised to receive one of two prophylactic regimes against deep vein thrombosis (DVT). These were 5000 units of subcutaneous heparin twice a day, alone or combined with the wearing of graded compression stockings. The diagnosis of DVT was made clinically and with ^131^I labelled fibrinogen. Six DVTs developed in the stocking group and 11 in the non-stocking group. The results suggest that the use of stockings reduces the incidence of DVT when added to herparin but the difference is not statistically significant. To obtain a predictive index for the development of DVT, discriminant analysis was applied to the control and stocking groups separately and combined. Five simple clinical variables gave a true positive prediction rate, for the combined group, of 94% and a false positive prediction rate of 26%.


### A Predictive Index for Post Operative Deep Vein Thrombosis in Thoracic Surgery Patients

**R B Richardson*, C Dufresne†, G E Staddon††, F A Chudry§, P Whitehead**, K Jeyasingham†.**
*Department of Medical Physics,
†Department of Thoracic Surgery,
**Department of Haematology,
Frenchay Hospital, Bristol.
††Department of Medical Physics, Bristol General Hospital.
§Computer Centre, Bristol Polytechnic, Bristol.

### ABSTRACT

In a single-centre prospective trial 200 consecutive patients undergoing thoracic surgery were randomised to receive one of two prophylactic regimes against deep vein thrombosis (DVT). These were 5000 units of subcutaneous heparin twice a day, alone or combined with the wearing of graded compression stockings. The diagnosis of DVT was made clinically and with $^{131}$I labelled fibrinogen. Six DVTs developed in the stocking group and 11 in the non-stocking group. The results suggest that the use of stockings reduces the incidence of DVT when added to heparin but the difference is not statistically significant. To obtain a predictive index for the development of DVT, discriminant analysis was applied to the control and stocking groups separately and combined. Five simple clinical variables gave a true positive prediction rate, for the combined group, of 94% and a false positive prediction rate of 26%.

### KEYWORDS

Thoracic Surgery 2) Deep vein thrombosis 3) Prophylactic regimes 3) $^{131}$I-fibrinogen 5) Stockings not statistically significant 6) Predictive index

### INTRODUCTION

The development of deep vein thrombosis (DVT) is a well known complication after any type of surgery. Various prophylactic regimes have been devised including the use of subcutaneous heparin (Kakkar et al 1972) and graded compression stockings (Holford et al 1976, Torngren et al 1980). Since 1970, low dose subcutaneous heparin (LDSH) has been shown to be effective in reducing the incidence of post operative DVT and subsequent fatal pulmonary embolism (PE) (International Multicentre Trial 1975). In spite of using low dose subcutaneous heparin there is still a significant number of patients with morbidity and mortality resulting from DVT and its sequelae. This study was designed to show whether wearing graded compression stockings in addition to the use of low dose subcutaneous heparin further reduces the incidence of DVT, and thus PE following thoracic surgery.

The protective role of LDSH having been proven (Kakkar et al 1972, International Multicentre Trial 1975) we felt that it would be unethical not to have all our patientss on heparin. Stockings are inconvenient and costly in time so a clear indication of their benefit is needed to justify their use. Indiscriminate application of prophylactic regimens involves unnecessary treatment of many patients who would not have developed DVT anyway. Some form of prediction of the patients who are at higher risk of developing DVT would, therefore, have considerable merit. Previous work on a predictive index has been published for gynaecological patients and involved special haematological investigations (Clayton et al 1976, Crandon et al 1980a & b). Our paper presents an index for thoracic surgical patients based solely on simple clinical parameters.

### PATIENTS AND METHODS

Two hundred consecutive patients, (135 males, 65 females) aged over 18, were randomly allocated to two groups. The control group received low dose subcutaneous heparin alone, while the stocking group received heparin and wore graded compression stockings. All patients received 5000 units of heparin with their pre-medication and 12 hourly thereafter for four days, or until fully mobile if that was longer. The thigh length stockings (TED, Kendall Co.) were fitted the day before operation and worn continuously until the end of the study. All patients underwent routine physiotherapy encouraging gentle exercise while in bed and early ambulation where possible.

### Clinical Data

The following clinical data were obtained: age; weight; height; sex; smoking habits; presence of varicose veins and history of venous thromboembolic disease (Pre-VTED). The male patients' desirable weight was calculated using the empirical formula:

$$\text{desirable weight (kg)} = 22.5 \times \text{height (m)}^2$$

The females' desirable weight was taken as five kilogrammes less than for males of the same height. Desirable weight calculated by this means compares well with that quoted in Documenta Geigy for persons of medium frame. The desirable weight subtracted from the patient's weight gave amount 'overweight' in kilogrammes. Also recorded were the number of days the patient was confined to bed before and after surgery; as was whether the operation was major or minor, the region (lung or others), and blood loss. Haematological analysis was carried out and included fibrin degradation products, activated partial thromoplasmin time, British comparative ratio, white cell count, haemoglobin, haematocrit and platelet counts.

### Diagnosis of DVT

i) $^{131}$I-fibrinogen scanning

DVT was diagnosed by the $^{131}$I-fibrinogen test with 3.7 megabequerel (MBq) given on the day before surgery and if a low count rate made it necessary, another 3.7 MBq was given about one week later. Using the technique of Negus et al 1968, the patients were scanned preoperatively then on alternate days, excluding Sunday, for 14 days or until discharge from hospital if that was sooner. DVT was diagnosed when the counts rose by 20% and was maintained for at least 24 hours. To protect the thyroid gland, doses of 150 mg of potassium iodide were given preoperatively and then daily throughout the course of the study.

ii) Clinical examination

This was based upon individual assessment of pain in the leg, local tenderness, oedema, dilated superficial veins and elevated skin temperation.

iii) Ascending venography

This was limited to those cases where there was a definite clinical diagnosis with a negative fibrinogen scan.

Statistical Analysis

The Statistical Package for the Social Sciences or SPSS (Nie et al 1975, Hull and Nie 1981) was used to provide frequency distributions, statistical and discriminant analyses for a variety of sub-groups and of the total data. Missing values were omitted from the analysis of that particular variable. Non-significance (n.s.) is taken when $p>0.050$. During discriminant analysis a list of variables is presented to the computer for possible inclusion in the discriminant equation. A step-wise approach is used whereby a variable is added to the discriminant function on the basis of maximising the distance between the group means. Positive values of the function imply a high risk of DVT, and conversely, negative values a low risk. Compromise between adequate sensitivity and poor specifity is embodied in the constant factor of the equation.

### RESULTS

Comparison of control and stocking groups.

There were 100 patients in each of the control and stocking groups and these were well matched for all the clinical and haematological variables investigated.

**Figure 1**

Histograms showing the day of onset of DVT for control and stocking groups.

Incidence of DVT

Of the 200 patients entered in the trial 38 were excluded from the DVT analysis. Of these eleven failed to receive heparin, 16 died before 14 days and 11 had incomplete scanning data. Sixteen patients developed a DVT as diagnosed by $^{131}$I-fibrinogen and a seventeenth DVT was included where a patient had a negative $^{131}$I-fibrinogen scan, clinical evidence of a PE and a positive venogram. Thrombosis developed in 11 of the 78 in the control group (14%) and in 6 of the 84 in the stocking group (7%). This difference in frequency is not statistically significant ($\chi^2=1.41$, $p>0.20$).

Considering the $^{131}$I-fibrinogen positive cases, the mean interval from surgery to the development of DVT was 5.2 days for the control group and 6.5 days for the stocking group. Seven of the ten patients in the control group and four of the six in the stocking group were receiving heparin when the clot was first detected (figure 1).

Initial analysis

Preoperative variables showing a significant positive association with DVT for all patients were age, pre-VTED and the presence of varicose veins (Tables 1 and 2). There was a negative association association between the platelet count and DVT. The only operative factor that was significant was operation site which indicated that DVT was less prevalent after operations on the lung. The variables associated with DVT for the control and stocking groups separately are also shown in Tables 1 and 2. All other clinical and haematological factors were found to be non-significant.

Discriminate analysis

Ten variables were considered for inclusion in the discriminant analysis for DVT. These included all variables significantly associated with DVT in any group, i.e. age, weight, haematocrit, platelets, pre-VTED, presence of varicose veins and operation site. Sex of the patient, severity of the operation and presence of malignancy were also included since these factors are generally accepted as influencing the development of DVT. Early runs showed that age over 40 allowed better prediction than age alone. Subsequent work employed this modified variable with those aged under forty scoring zero and those over this age having forty subtracted from their age. Similarly overweight was used instead of the patient's actual weight. The best discrimination was obtained when the variables used were (a) pre-VTED, (b) age over 40, (c) overweight, (d) sex and (e) presence of varicose veins whether the group were analysed separately or combined.

**Figure 2**

Histogram of predictive indices (I) for all patients.

**Table 1**

**Continuous Preoperative Data having a Significant Correlation with the Development of DVT. Units for Age, Weight, Haematocrit and Platelets are Respectively Year, Kilogram, Ratio and $10^9$/litre**

| | *Variables* | *DVT group mean & st.dev.* | *non-DVT group mean & st.dev.* | *significance (students' t)* |
|---|---|---|---|---|
| CONTROL GROUP | Age | 71+11 | 60+13 | p<0.005 |
| | Weight | 65+14 | 64+12 | n.s. |
| | Haematocrit | 0.38+0.04 | 0.40+0.05 | n.s. |
| | Platelets | 280+120 | 340+140 | n.s. |
| STOCKING GROUP | Age | 67+6 | 59+14 | p<0.01 |
| | Weight | 76+9 | 66+12 | p<0.02 |
| | Haematocrit | 0.43+0.02 | 0.41+0.05 | p<0.05 |
| | Platelets | 220+46 | 310+130 | p<0.001 |
| COMBINED GROUP | Age | 69+9 | 59+13 | p<0.001 |
| | Weight | 69+13 | 65+12 | n.s. |
| | Haematocrit | 0.40+0.04 | 0.41+0.05 | n.s. |
| | Platelets | 260+100 | 330+130 | p<0.010 |

**Table 2**

**Dichotomous preoperative data having a significant correlation with DVT**

| | *Variables* | *DVT group* | *non-DVT group* | *Significance (Chi-square)* |
|---|---|---|---|---|
| CONTROL GROUP | Pre-VTED | 2/11 (18%) | 0/67 (0%) | p<0.02 |
| | Varicose veins | 5/11 (45%) | 19/54 (35%) | n.s. |
| | Operation site | 4/11 (36%) | 40/67 (60%) | n.s. |
| STOCKING GROUP | Pre-VTED | 1/6 (17%) | 3/98 (4%) | n.s. |
| | Varicose veins | 5/6 (83%) | 16/68 (24%) | p<0.01 |
| | Operation site | 2/6 (33%) | 53/78 (68%) | n.s. |
| COMBINED GROUP | Pre-VTED | 3/17 (18%) | 3/145 (2%) | p<0.020 |
| | Varicose veins | 10/17 (59%) | 35/122 (29%) | p<0.030 |
| | Operation site | 6/17 (35%) | 93/145 (64%) | p<0.050 |

The best discriminant function for the eleven DVTs in the control group, gave a 91% true positive and a 35% false positive prediction rate. For the six patients with DVT in the stocking group there was an 83% true positive and a 13% false positive prediction rate. As the number of DVTs in these two groups is small and since there was no significant difference in incidence between the groups, we also performed discriminant analysis on both groups combined. In this case the index was:-

$$I=0.059a+0.036b+3.79c+0.68d+0.77e-2.24$$

This predicted 16 of the 17 clots (94%) with a false positive rate of 32 out of 122 (26%). A histogram of the values of the index for all cases with complete data for the five variables chosen is shown in figure 2.

### DISCUSSION

The overall incidence of DVT in this series was 10% (17 of 162 patients). We have not been able to show a statistically significant improvement in DVT prophylaxis arising from the additional use of graded compression stockings although the incidence was reduced to 7% compared with 14% in the control group. If this improvement is real then a larger series of patients must be investigated, a total of 700 patients would give a 75% chance of arriving at a 'significant' result (Boag et al 1971).

Four patients, none of whom wore stockings, were diagnosed clinically as having had a PE. In the three who died the diagnosis was confirmed at post mortem examination. The fourth patient recovered with treatment. Three of these patients had negative $^{131}$I-fibrinogen scans. Samples of clots from one of them showed a negligible radioactivity. The topping-up of $^{131}$I-fibrinogen was designed to overcome this problem which was attributed to the concentration of radiopharmaceutical in the site of any haematoma in the thoracotomy wound. Of the six patients with a previous history of DVT both of those in the control group developed a further DVT whilst only one of the four in the stocking group did so.

Comparison of the clinical and fibrinogen uptake findings showed that in the control group five out of the ten $^{131}$I-fibrinogen positive cases were also diagnosed clinically whereas in the stocking group only one of the six cases was diagnosed. With such small numbers this difference is not statistically significant but indicates that wearing stockings may interfere with the clinical diagnosis of DVT.

Initial analysis of the all patient data (Tables 1 and 2) identified significant correlations with DVT for age, history of venous thromboembolic disease, and varicose veins which has been found in previous studies. There was also a negative correlation with the pre-operative platelet count; the reason for this is not clear. The sex of the patient and whether there was benign or malignant disease present were not significant.

The second objective of this study was to find a simple yet effective form of prognostic index. Age over 40 years was found to be more useful than age itself, because of the non-linear relationship between age and the incidence of DVT. Similarly 'overweight' proved to be a better predictor of DVT than the patient's actual weight. Although the sex of the patient was not significantly associated with the occurrence of DVT it was consistently selected by the computer as a useful variable for the discriminant function. It is notable that the sign of the coefficient means that being male produces an increase in the prognostic index implying an increased risk of DVT. Our aim was to derive predictive indices for the control and stocking groups separately but the number of DVTs was too few to do this properly. Correlations of variables with DVT for the separate groups indicate that the index for the combined group is not ideal and that separate indices might be more powerful, but, a larger trial would be necessary to prove this.

The discriminating power of predictive index for the combined group is comparable with that of Clayton et al 1976, but uses simple clinical factors only, making it suitable for a pocket-calculator or computerised patient admission system.

### ADDENDUM

Our policy since this study is now to use subcutaneous Heparin routinely in all patients, and in the high risk (high predictive index) group we use Heparin and graded stockings.

### ACKNOWLEDGEMENTS

We thank Mr A. Al-Jilaihawi, Department of Thoracic Surgery, Frenchay Hospital, for his help in initiating this study. We are grateful to Mr D. Gethin and Mr J. Gowers of Bristol Polytechnic for their help and advice with the computing and statistical analysis. We would like to thank Dr M. Sweeney for helping with the collection of patient data.

### REFERENCES

BOAG, J. W., HAYBITTLE, M. A., FOWLER, J. F. and EMERY, E. W. (1971) *Br.J.Surg.* **44**, 122–25.

CLAYTON, J. K., ANDERSON, J. A. and McNICOL, G. P. (1976) *Br.Med.J.* **ii**, 910–2.

CRANDON, A. J., PEEL, K. R., ANDERSON, J. A., THOMPSON, V. and McNICOL, G. P. (1980a) *Br.Med.J.* **ii**, 343–4.

CRANDON, A. J., PEEL, K. R., ANDERSON, J. A., THOMPSON, V. and McNICHOL, G. P. (1980b) *Br.Med.J.* **ii**, 345–7.

Documenta Geigy Scientific Tables 7th ed (1970) (Macclesfield: Geigy Pharmaceuticals).

HOLFORD, C. P. (1976) *B.Med.J.* **2**, 969–70.

HULL, C. H. and NIE N. H. (1981) SPSS update 7–9 (Hightstown: McGraw-Hill) 292–300.

International Multicentre Trial (1975) *Lancet* **ii**, 45–51.

KAKKAR, V. V., CERRIGAN, T., SPINDLER, J., FOSSARD, D. P., FLUTE, P. T., CRELLIN, R. Q., WESSLER, S. and YIN, E. T. (1972) *Lancet* **ii**, 101–6.

NEGUS, D., PINTO, D. J., QUESNE, L. P., BROWNE, N. and CHAPMAN, M. (1968) *Br.J.Surg.* **55**, 835–39.

NIE, N. H., HULL, C. H., JENKINS, J. G., STEINBRENNER, K. and BENT D. H. (1975) Statistical package for the social sciences 2nd ed. (Hightstown: McGraw-Hill) 434–67.

TÖRNGREN, S. (1980) *Br.J.Surg.* **67**, 482–4.

## Figures and Tables

**Figure 1 f1:**
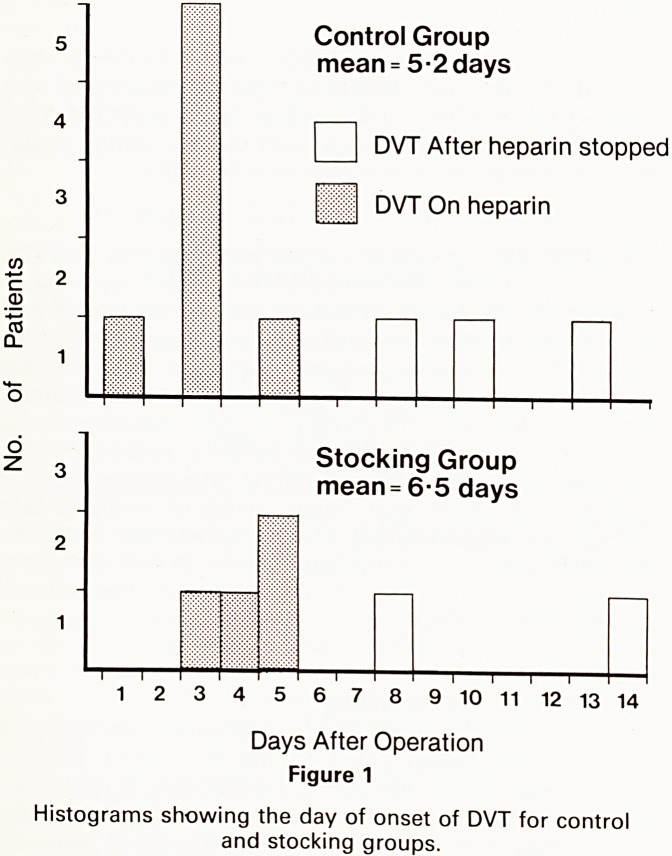


**Figure 2 f2:**